# Molecular Mechanisms of Getah Virus Pathogenesis

**DOI:** 10.3390/vetsci13101092

**Published:** 2026-10-15

**Authors:** Muhammad Umar Sharif, Chunchun Liu, Yafeng Qiu

**Affiliations:** Shanghai Veterinary Research Institute, Chinese Academy of Agricultural Science, Shanghai 200241, China

**Keywords:** GETV, viral pathogenesis, alphavirus, tissue tropism, host–virus interactions, immune evasion, host adaptation

## Abstract

Getah virus (GETV), a mosquito-borne alphavirus within the *Togaviridae* family, produces distinct pathological outcomes in different hosts, highlighting that viral and host molecular interactions determine disease phenotypes. For instance, horses typically develop acute febrile disease accompanied by limb edema and cutaneous rash, whereas pigs may experience reproductive failure, fetal infection, and severe neonatal disease. These host-dependent outcomes indicate that GETV pathogenesis involves the combined effects of viral entry, intracellular replication, host antiviral responses, tissue permissiveness, and viral dissemination. The E1/E2 envelope glycoproteins contribute to cellular attachment, membrane fusion, and viral entry, while the non-structural proteins nsP1–nsP4 coordinate viral RNA replication and interact with the host’s cellular pathways. Among these proteins, nsP2 contributes to suppression of antiviral responses via TBK1–IRF3 and JAK–STAT signaling, whereas nsP3 participates in replication complex formation and interactions with host factors by disruption of G3BP1-associated stress granules. Genetic variation among GETV lineages is also associated with differences in epidemiology and pathogenic strains, although the causal contribution of individual mutations remains incompletely defined. Functional studies of envelope variation, including the E2-N262 glycosylation site, further indicate that viral surface properties can influence replication and virulence. Despite increasing molecular and epidemiological evidence, important gaps remain in understanding the relative contribution of different entry factors, cellular permissiveness, host-dependent pathogenicity, and the functional consequences of naturally occurring viral variation. This review integrates the current evidence on GETV viral proteins, replication, cellular entry, host antiviral responses, tissue tropism, host-specific pathology, and viral genetic diversity to establish the current molecular framework of GETV pathogenesis and highlight the priorities for future experimental validation and disease control.

## Simple Summary

Getah virus (GETV) is an emerging mosquito-borne virus that can infect several animal species and causes distinct pathogenesis among hosts. This review brings together current evidence on how viral entry, replication, immune evasion, tissue permissiveness, and vector–host interactions contribute to these differences. We highlight that GETV pathogenesis is multifactorial and cannot be explained by viral genetic variation alone. Although important advances have been made, the relative contribution of different entry factors, mechanisms underlying host-specific tissue tropism, and the functional effects of viral variation remain incompletely understood. Addressing these gaps will support improved surveillance, vaccines, and disease-control strategies.

## 1. Introduction

Getah virus (GETV) is an emerging mosquito-borne alphavirus in the family *Togaviridae* [1], that circulates in complex enzootic transmission cycles involving multiple mosquito vectors (approximately 17 species from five *Culicidae* genera, including *Culex, Anopheles, Armigeres, Aedes* and *Mansonia*) and a broad range of vertebrate hosts. Its increasing geographic expansion and host diversification have elevated GETV into an important veterinary and public health concern across Asia [2]. GETV was first isolated from *Culex gelidus* mosquitoes in a Malaysian rubber plantation in 1955 [3]. Since then, the virus has been identified across tropical, subtropical, and temperate regions, ranging from Southeast Asia to East Asia, the Russian Far East, and Australia, although no confirmed circulation has been reported in Europe, Africa, or the Americas. Serological surveillance demonstrates that GETV is now endemic in many parts of Eurasia, with particularly high circulation in domestic livestock populations in several Asian countries. The variability in regional seroprevalence likely reflects differences in mosquito ecology, climatic conditions, agricultural practices, and land-use patterns [4,5].

GETV has been detected in a wide variety of vertebrate hosts, including horses [6], pigs [7], cattle [8], blue foxes [9], red pandas [10], wild boars [11], and humans [2]. Horses and pigs are considered major amplification hosts that contribute substantially to viral maintenance and transmission [12,13]. Clinically, GETV infection exhibits pronounced host-dependent pathogenicity. Horses typically develop acute febrile disease accompanied by limb edema, cutaneous rash, and lymphadenopathy [2,14], whereas pigs frequently exhibit reproductive failure, abortion, fetal death, and high neonatal mortality [15].

It is worth noting here that the broad host range, together with repeated GETV outbreaks in Japan, China, and India, has raised serious concerns about its potential as an emerging livestock pathogen, especially in regions with abundant vector populations. Although serological evidence has suggested previous human exposure, no confirmed clinical disease attributable to GETV has been established [2,16,17]. Furthermore, human-derived cell lines have shown resistance to GETV infection in laboratory studies [18]. Thus, GETV remains primarily an animal pathogen, and its zoonotic potential and public health significance require further investigation.

The same virus can induce distinct, tissue-specific pathological changes in different host species [19]. These clinical outcomes highlight that GETV pathogenesis is not only dependent on viral replication but also on the interactions among viral entry processes, intracellular replication, host immune response, cellular permissiveness, and tissue microenvironment, along with viral dissemination [19,20]. Together, these factors can contribute to the multifactorial phenotype of GETV tissue tropism.

However, despite increasing epidemiological and molecular evidence, the mechanisms underlying GETV host specificity, tissue tropism, and immune evasion remain incompletely understood [15]. Important gaps persist in understanding the molecular processes governing viral entry following attachment, cellular permissiveness, modulation of host antiviral response, and how genetic variation influences pathogenicity [21,22]. Therefore, this review synthesizes the available evidence on the molecular mechanisms underlying GETV pathogenesis, focusing on viral entry, host–pathogen interactions, immune evasion, host-specific pathogenesis, and genetic variation, while highlighting areas that need further experimental validation.

## 2. Taxonomy, Genome Organization, and Viral Proteins

### 2.1. Taxonomic Perspective

Alphaviruses are positive-sense RNA, enveloped viruses and are broadly classified into Old-World and New-World alphaviruses based on their epidemiological characterization, genomic relationships, and serological properties. Old-World alphaviruses, including Sindbis virus and Semliki Forest virus, are typically associated with arthritogenic diseases, whereas several New-World alphaviruses, such as Venezuelan, Eastern, and Western equine encephalitis viruses, are predominantly associated with neurological symptoms [23]. GETV is an Old-World alphavirus that exhibits an arthritogenic phenotype, with musculoskeletal and inflammatory manifestations reported in infected hosts [24]. However, GETV pathogenesis extends beyond joint and musculoskeletal involvement and is strongly host-dependent. In particular, infection in pigs has been associated with reproductive failure, fetal infection, abortion, stillbirth, and severe neonatal disease [25], while neurological manifestations have also been reported in piglets [17]. Thus, although GETV shares the arthritogenic characteristics of several Old-World alphaviruses, its multi-host pathogenesis encompasses reproductive, systemic, and neurological manifestations in addition to musculoskeletal disease.

### 2.2. Genomic Characterizations

**Figure 1:**
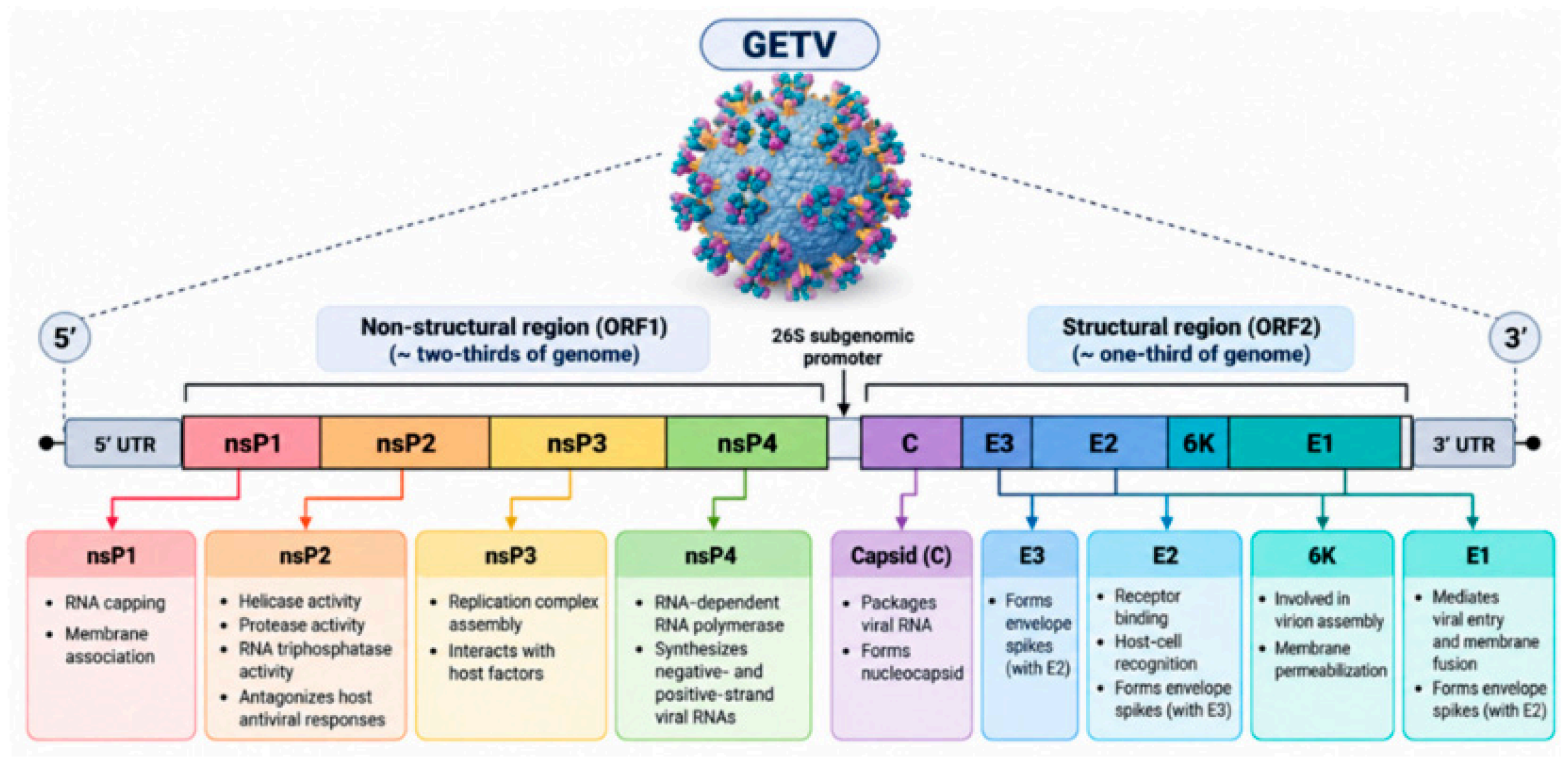
**Genomic organization of GETV:** The GETV genome is organized into two open reading frames (ORFs), ORF1 and ORF2, where ORF1 occupies 2/3 of the genome to encode non-structural proteins, and ORF2 occupies the remaining 1/3 to translate structural proteins. Four non-structural proteins (nsP1-nsP4) are involved in viral replication and host cell interactions. Five structural proteins (C, E3, E2, 6K, E1) are responsible for viral particle assembly and host cell invasion. The figure was designed using Adobe Photoshop 2020.

GETV is a single-stranded positive-sense RNA virus belonging to Old-World alphaviruses, having an approximately 11–12 kb genome with 5′ methylated cap (7-methylguanosine) and 3′ polyadenylated tail [2]. The GETV genome is organized into two open reading frames (ORFs). ORF1, located towards the 5′ end, encodes the non-structural proteins and covers approximately 2/3 of the whole genome. The remaining 1/3 contains ORF2, which encodes the structural proteins. An internal 26S subgenomic promoter is located upstream of ORF2 and directs the transcription of subgenomic RNA encoding the structural proteins (Figure 1) [12,15]. Furthermore, it is believed that certain genomic regions, such as untranslated regions (UTRs) and Poly (A) tail, contribute to RNA stability and translation efficiency [13].

The non-structural proteins (nsP1–nsP4) form a replication complex on cytoplasmic membranes (Table 1). nsP1 mediates RNA capping and membrane association, nsP2 influences RNA processing to antagonize host antiviral responses, nsP3 supports assembly of the replication complex and host factor interactions, and nsP4 functions as the RNA-dependent RNA polymerase [2,17]. The structural proteins E1 and E2, together with E3, form envelope spikes involved in viral entry and host cell recognition, while the capsid protein packages the viral genome into the nucleocapsid [12]. Thus, the non-structural proteins primarily support RNA replication and host response modulation, whereas the structural proteins mediate entry, assembly, and virion formation [26].

### 2.3. Functional Role of Viral Structural Proteins

Structural proteins of GETV coordinate viral attachment, entry, assembly, and release. E2 plays an important role in viral attachment and entry via host entry receptor binding and involving cellular attachment factors [21], whereas E1, a class II fusion protein, supports low-pH-dependent membrane fusion in endosomes, enabling nucleocapsid delivery into the system [27]. The capsid protein packages viral RNA [28], while the 6K protein and its trans-frame product contribute in cellular ion channel activity, efficient glycoprotein trafficking, and ultimately virion budding, leading to viral particle stability and release (Table 2) [29]. Although these surface glycoproteins are essential for viral attachment, entry, and particle formation, tissue tropism results from their interaction with cellular permissiveness, host factors, and tissue-specific factors [12]. Together, these proteins support the viral life cycle, while their interactions with host factors may influence GETV pathogenesis [21].

**Table 1:** Summary of the functions of GETV viral proteins.

ORFs	Protein	Main Functions	References
ORF1/Non-structural Proteins	nsP1 nsP2 nsP3 nsP4	Formation of viral replication complex for the synthesis of genomic and sub-genomic viral RNA needed for downstream structural protein synthesis. Individual proteins perform distinct functions: (1) RNA capping (nsP1). (2) RNA processing (nsP2). (3) Replication complex assembly (nsP3). (4) RNA synthesis (nsP4). and may contribute to host interactions and virulence.	[2,17,30]
ORF2/Structural Proteins	Capsid (C)	(1) Forms the nucleocapsid around the viral RNA. (2) Protects and packages the viral genome during particle formation.	[28]
	E1, E2	(1) E1/E2 make heterodimers in trimeric form. (2) Support viral attachment and entry during infection. (3) Principal targets for neutralizing antibodies.	[27,30]
	E3	(1) Assists the folding and maturation of envelope glycoproteins. (2) Supports envelope spike assembly. (3) Helps stabilize the E1–E2 complex and prevents premature conformational changes.	[17,31]
	6K	(1) Facilitates the release of newly assembled viral particles. (2) Supports E2 transport from the Golgi apparatus to the plasma membrane. (3) Contributes to GETV replication and pathogenicity.	[29]

## 3. Molecular Mechanisms of Viral Replication

### 3.1. Role of Non-Structural Proteins

**Figure 2:**
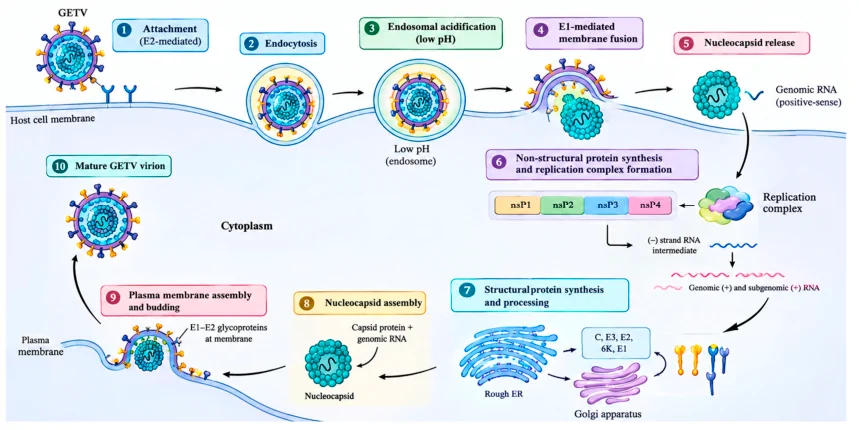
**Replication cycle of GETV in host cells.** GETV initially attaches to host cell surface factors through the viral E2 glycoprotein and is internalized through endocytosis. Acidification of the endosomal compartment induces conformational rearrangement of the E1–E2 complex, leading to E1-mediated membrane fusion and release of the viral nucleocapsid into the cytoplasm. The viral genomic RNA is translated to produce the non-structural polyprotein, which is processed into nsP1–nsP4 and forms the viral replication complex. This complex synthesizes negative-strand RNA intermediates and subsequently genomic and subgenomic positive-sense RNAs. The subgenomic RNA directs synthesis of the structural proteins C, E3, E2, 6Kand E1. Viral glycoproteins undergo processing and maturation through the endoplasmic reticulum and Golgi apparatus, while the capsid protein associates with genomic RNA to form nucleocapsids. The viral structural components are transported to the plasma membrane, where nucleocapsids interact with membrane-associated E1–E2 glycoproteins and assemble into mature virions that bud from the plasma membrane [38]. The figure was designed using Adobe Photoshop 2020.

Non-structural proteins are translated as a polyprotein that undergoes proteolytic processing to make nsP1-nsP4, which form a replication complex (Figure 2). Each protein performs a distinct function and coordinates with other proteins for replication and host immune interaction (Table 2). Beyond their direct role in RNA synthesis, the non-structural proteins modify the intercellular environment to interact with the host cellular pathways that support viral replication and influence antiviral responses [24,32].

nsP1 is a membrane-associated alphavirus protein involved in viral RNA capping, with experimentally demonstrated methyltransferase and guanylyltransferase activities. This membrane-binding activity also helps to organize the replication complex on intercellular membranes, providing a platform for viral RNA synthesis [33]. In GETV, nsP1 is predicted to perform the same function in viral RNA capping, although direct biochemical confirmation of its enzymatic activities should be distinguished from evidence obtained from other alphaviruses.

nsP2 contains helicase, protease, and NTPase to support viral RNA replication and polyprotein processing. GETV-specific studies have demonstrated that nsP2 antagonizes host interferon responses through mechanisms involving IRF3 signaling and inhibition of IKKε-associated antiviral activity [26,34]. These functions may facilitate viral replication by reducing host antiviral response, although the precise contribution of individual nsP2 activities to GETV pathogenesis requires further investigation.

nsP3 acts as a multifunctional adaptor that can recruit various host proteins to support replication complex formation, as described in alphaviruses [35]. In GETV, nsP3 acts as a critical modulator that interacts with G3BP and promotes the formation of aggregates that differ from bona fide stress granules [36]. This interaction alters the normal organization of G3BP and may influence the cellular environment during infection; however, its precise contribution to GETV replication depends on the experimental context.

nsP4, the RNA-dependent RNA polymerase, catalyzes viral RNA synthesis, including the production of negative-strand templates and new genomic and subgenomic RNAs. Studies of alphaviruses have characterized nsP4’s dynamic polymerase architecture [37], while GETV reviews have described its role in viral RNA replication as RNA-dependent RNA polymerase [38]. Nevertheless, detailed biochemical characterizations of GETV nsP4 should be distinguished from findings established based on other alphaviruses. Thus, nsP1-nsP4 constitutes a fully functional replication machinery that can promote viral RNA synthesis and engagement of antiviral host pathways.

### 3.2. Host Cell-Associated Factors Supporting Replication

Host cell-associated factors promote efficient GETV replication by contributing to viral entry, formation of the replication complex, and remodeling of virus-induced membranes. GETV, like other alphaviruses, utilizes the E2 glycoprotein to interact with cellular attachment factors, including heparan sulfate, facilitating viral attachment [39]. Following attachment, alphaviruses generally enter cells through endocytosis, while intracellular membrane organization and trafficking support the formation of replication sites [40]. However, the extent to which these processes have been directly characterized in GETV remains poorly understood.

At the molecular level, GETV interacts with host proteins involved in translation, stress responses, and RNA metabolism. Therefore, nsP3 interacts with G3BP to promote the formation of aggregates that differ from bona fide stress granules, potentially altering cellular antiviral responses [36]. Thus, the host membrane composition and intracellular organization may further influence viral replication and virion production [40]. These findings suggest that differences in host cell compatibility may contribute to variations in GETV replication across cell types and influence tissue-specific permissiveness. Nevertheless, the relative contribution of these host factors to GETV tissue tropism requires further experimental validation [36].

**Table 2:** Synthesis of viral protein interactions and molecular functions involved in GETV pathogenesis.

Viral Protein	Host Factor/Interaction	Principal Mechanism/Consequence	References
E1	E1–E2 complex; host cell membrane	Low-pH-mediated membrane fusion and nucleocapsid release	[12,27,38,41]
E2	Heparan sulphate; LDLR, MXRA8	Cell attachment, receptor-mediated entry and modulation of viral fitness	[21,22,39,42]
6K/TF	Host cellular membranes	Glycoprotein trafficking and virion budding	[29,43]
Capsid (C)	Viral genomic RNA; E1–E2-containing membrane	Nucleocapsid formation and virion assembly	[27,28]
nsP1	Membrane-associated replication machinery	Establishment of membrane-associated sites for viral RNA synthesis	[33,38]
nsP2	TBK1/IRF3; IKKε	Suppression of interferon production/signaling and host transcriptional shut-off	[26,34,44,45]
nsP3	G3BP1	Modulation of stress granule formation and promotion of viral replication	[35,36]
nsP4	nsP1–nsP3 replication machinery	Synthesis of genomic and subgenomic viral RNA	[37,38]

## 4. Molecular Mechanisms of Cell Tropism and Viral Entry

### 4.1. Envelope Glycoprotein (E1 and E2)

Envelope glycoproteins E1 and E2 appear on the virion surface (as heterodimeric spikes) and coordinate receptor binding along with membrane fusion for early-stage viral entry. High-resolution cryo-EM has revealed that these heterodimeric spikes form a specialized icosahedral lattice in GETV. In this lattice, E2 is located at the periphery to interact with host receptors; however, E1 acts as a fusion protein (class II) that becomes activated in the low pH of endosomes [12]. Thus, E2 contributes to viral attachment, and E1 mediates membrane fusion for successful viral entry. Therefore, alterations in either process may influence cellular and tissue tropism.

GETV entry involves coordinated interactions between the viral E1–E2 glycoprotein complex and host cell surface factors. Heparan sulphate functions as an attachment factor that facilitates initial virion binding, whereas the low-density lipoprotein receptor (LDLR) has been experimentally demonstrated to contribute to GETV receptor engagement and productive entry [21,22,39]. Structural features of E2, including surface-exposed residues and N-linked glycosylation sites, can modulate these interactions and consequently influence viral attachment, entry, and fitness.

Recent studies have reported a conserved N-glycosylation site in E1 (N141) and E2 (N200, N262), which are assumed to modulate receptor attachment and conformational rearrangements that are required for virion fusion. Moreover, experimental studies have highlighted envelope glycosylation as a functional determinant of viral stability rather than only a structural modification due to its influence on GETV replication, virulence, and host immune recognition [15,21].

After attachment, E1–E2 complexes enter into cells via endocytosis, and endosomal low pH (acidic environment) influences the dissociation of the heterodimer, leading to E1 fusion loop exposure along with insertion within the host membrane [41]. This E1 refolding (pH-dependent) into a homotrimer allows for nucleocapsid release into the cytoplasm and initiates GETV infection [12].

### 4.2. Host Receptors and Attachment Factors

Recent experimental evidence has established a role for the low-density lipoprotein receptor (LDLR) in GETV entry. A 2026 study demonstrated that the GETV E2 glycoprotein interacts with LDLR and that E2 residue 86 contributes to both glycosaminoglycan- and LDLR-dependent infection [22]. Further evidence showed that GETV envelope glycosylation can modulate LDLR binding and entry, with the E2-N262 glycan enhancing the interaction with LDLR and contributing to viral fitness in mammalian cells [21]. These findings provide direct evidence that LDLR functions as a GETV entry receptor rather than merely representing a receptor candidate inferred from related alphaviruses. In addition to LDLR, MXRA8 was identified as a host entry receptor for alphaviruses, including GETV. Knockout and receptor-restoration experiments in BHK-21 cells support its contribution to GETV infection, although its relative importance across different host species and tissues remains to be established [46].

Heparan sulphate, an abundant sulfated glycosaminoglycan on the mammalian cell surface, should be distinguished from protein entry receptors. HS was experimentally identified as an attachment factor for GETV and contributes to the initial interaction of virions with host cells [22]. Substitution of E2 residue 253 with arginine (K253R) enhances HS binding and virion attachment in vitro, but reduces viral replication and fitness in vivo, demonstrating that increased attachment to cell-surface glycans does not necessarily result in enhanced systemic pathogenicity [39]. Thus, HS-mediated attachment and LDLR-mediated receptor engagement represent distinct but potentially interconnected stages of GETV entry. Similar effects of glycosaminoglycan interactions on alphavirus attachment were also reported for SINV and CHIKV [41,47]. However, the extent to which these comparative findings explain GETV tissue tropism requires further investigation.

This evidence supports a model in which GETV attachment can involve interactions with cell-surface glycosaminoglycans such as HS, followed by engagement of protein receptors, including LDLR. However, the relative contribution of these factors may vary according to the viral genotype, envelope glycosylation state, host cell type, and tissue context. Further studies using receptor-deficient cells, receptor complementation, and physiologically relevant animal models are required to determine how receptor usage contributes to GETV tissue tropism and host adaptation.

### 4.3. Viral Mutations Influencing Cellular Tropism

GETV displays significant genetic diversity among its phylogenetic groups (GI–GIV). Comparative genomics has found a distinct amino acid substitution in E2 and in the hypervariable region of nsP3 that is believed to influence host range and epidemic potential. For instance, the GIII strain (epidemic strain) is different from other non-epidemic strains on the basis of its multiple E2 sites, supporting receptor usage, stability of E1-E2 spikes, and antibody escape [48,49]. However, comparative association alone cannot establish that individual substitutions are responsible for altered tissue tropism or virulence; functional validation is required to distinguish lineage-associated markers from causal determinants.

Viral mutations can influence host interactions, tissue tropism, and pathogenicity, although these effects may differ among alphaviruses. In GETV, the E2 K253R substitution illustrates how enhanced attachment in vitro may not translate into increased viral fitness in vivo [39]. However, although genomic and reverse genetics studies have identified differences associated with viral fitness and disease severity, the extent to which individual mutations directly determine tissue tropism remains incompletely understood. Further comparative and functional studies are needed to establish the contribution of specific viral mutations to host adaptation and tissue-specific pathogenesis.

### 4.4. Envelope Glycosylation and GETV Fitness

N-glycosylation of GETV envelope proteins E1 and E2 is a significant factor in viral fitness, host adaptation, and pathogenicity. Conserved glycosylation sites are located within surface glycoproteins and could affect virus replication, host cell interactions, and immune recognition at E1-N141, E2-N200, and E2-N262 [12,21,50]. Recent reverse genetics studies have shown that the removal of these glycosylation sites has various effects on the phenotype, suggesting that individual glycans are not equally important for the fitness of GETV.

**Figure 3:**
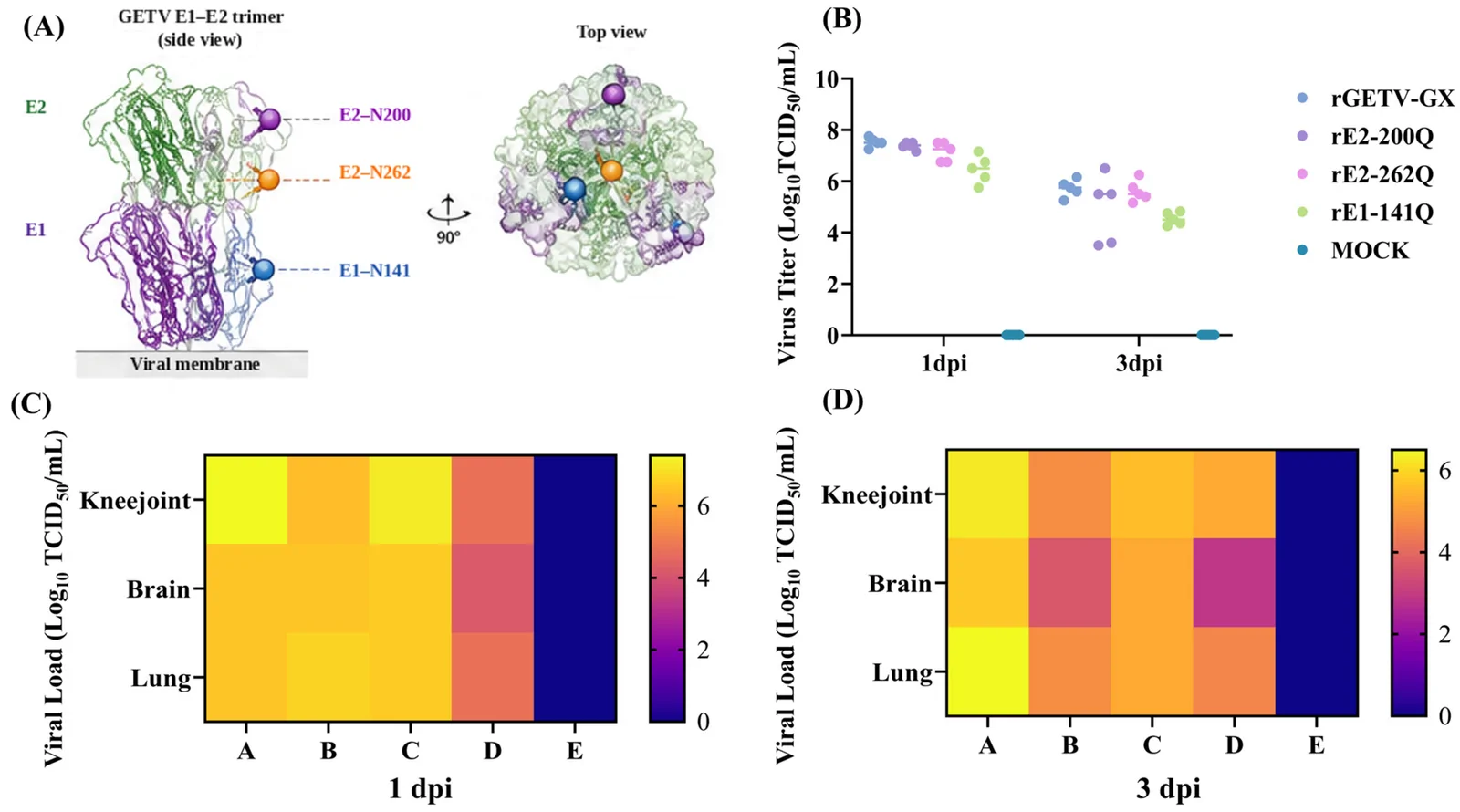
**Glycosylation influences GETV replication and tissue distribution.** (**A**) Schematic diagram showing location of E1-N141, E2-N200 and E2-N262 on the GETV E1–E2 complex. (**B**) Viral titers of recombinant GETV and glycosylation-site mutants at 1 and 3 dpi. where A =rGETV-GX, B =rE2-200Q, C = rE2-262Q, D = rE1-141Q, and E = MOCK (**C**,**D**) Tissue viral RNA levels in the knee joint, brain, and lung at 1 and 3 dpi, respectively. Heatmap values represent log_10_ viral RNA copies/g tissue. Data are replotted using GraphPad Prism 10.5.0 version, from Ren et al. (2026), PLOS Pathogens, 22, e1014126, under the Creative Commons Attribution (CC BY 4.0) license. No additional permission is required [50].

E2-N262 is one of these sites that has been the subject of special interest due to its glycosylation-dependent role in viral replication and host-dependent role in viral virulence. A change in E2-N262 was found to influence viral replication in mammalian cells and mosquitoes, and glycan-deficient viruses exhibited a decrease in pathogenicity in mouse models and an increase in sensitivity to neutralizing antibodies [21,50]. The results here indicate that E2-N262 could play a role in viral fitness as well as immune evasion and persistence of productive infection in vivo. Experimental substitutions of E1-N141, E2-N200 and E2-N262 altered viral replication in vivo, with mutant viruses showing distinct replication profiles at 1 and 3 dpi and differences in viral burden across the knee joint, brain and lung (Figure 3).

Conversely, envelope glycans may be important for additional roles in regulating the functional properties of the E1/E2 complex, such as those observed for modifications of E1-N141 and E2-N200, which impact viral replication and host-associated phenotypes [50]. Importantly, the effect of glycosylation varies among experimental systems. A mutation that boosts the ability of a cell culture to replicate or attach does not invariably boost in vivo virulence within the mammalian system, where different selective pressures and immune responses operate, as well as different mosquito vectors. In this light, envelope glycosylation represents an evolutionary tuning process that fine-tunes the viral entry, replication, immune recognition and fitness in various hosts. Taken together, the results suggest glycosylation at E1/E2 is a key molecular connection between viral variation and the GETV host- and tissue-specific phenotypes [21].

## 5. Host Immune Responses and Viral Evasion

Following cellular entry and initiation of replication, GETV encounters innate antiviral mechanisms that can restrict viral replication and dissemination. In response, GETV employs viral proteins that interfere with host antiviral signaling and cellular stress responses. The balance between antiviral restriction and viral immune evasion can influence the extent of viral replication, systemic dissemination, and tissue injury. Thus, host immune responses represent an important mechanistic link between intracellular viral replication and the host- and tissue-specific manifestations of GETV infection.

### 5.1. Host-Associated Innate Immune Responses

Type I interferon (IFN-I) responses constitute a major early antiviral defense against GETV infection. Cytosolic RNA sensors, including retinoic acid-inducible gene I (RIG-I) and melanoma differentiation-associated protein 5 (MDA5), can identify viral RNA replication intermediates, transmitting signals via mitochondrial antiviral-signaling protein (MAVS). The stimulation of MAVS enhances downstream kinase signaling that includes TANK-binding kinase 1 (TBK1) and IκB kinase ε (IKKε), resulting in the phosphorylation and activation of interferon regulatory factor 3 (IRF3). Activated IRF3 translocates to the nucleus and transactivates the expression of IFN-β and other antiviral genes [51,52]. Secreted interferons subsequently act in an autocrine and paracrine manner to establish an antiviral state in infected and neighboring cells.

The timing and magnitude of this response may influence whether replication of GETV remains locally controlled or progresses towards systemic dissemination. Consequently, cellular susceptibility to GETV depends not only on the viral attachment and entry, but also on the capacity of infected cells to mount an effective antiviral response. Differences in interferon responses among cell types may, therefore, contribute to variation in cellular and tissue permissiveness. The experimental evidence that exogenous IFN-γ can inhibit GETV replication further supports a role for interferon-mediated antiviral activity in restricting infection [53].

### 5.2. Mechanisms of Immune Evasion

GETV mitigates inherent antiviral signaling through multiple pathways, with nsP2 serving as a primary viral antagonist. GETV nsP2 impedes IFN-β synthesis by disrupting IRF3 activation. Recent mechanistic investigations revealed that nsP2 engages with TBK1 and inhibits the phosphorylation of IRF3, while simultaneously disrupting the association between phosphorylated IRF3 and the nuclear import proteins KPNA3 and KPNA4. As a result, the nuclear translocation of IRF3 and the production of IFN-β are diminished [26,34,45]. These strategies provide GETV with an initial method to suppress host antiviral signaling while permitting ongoing viral replication.

**Figure 4:**
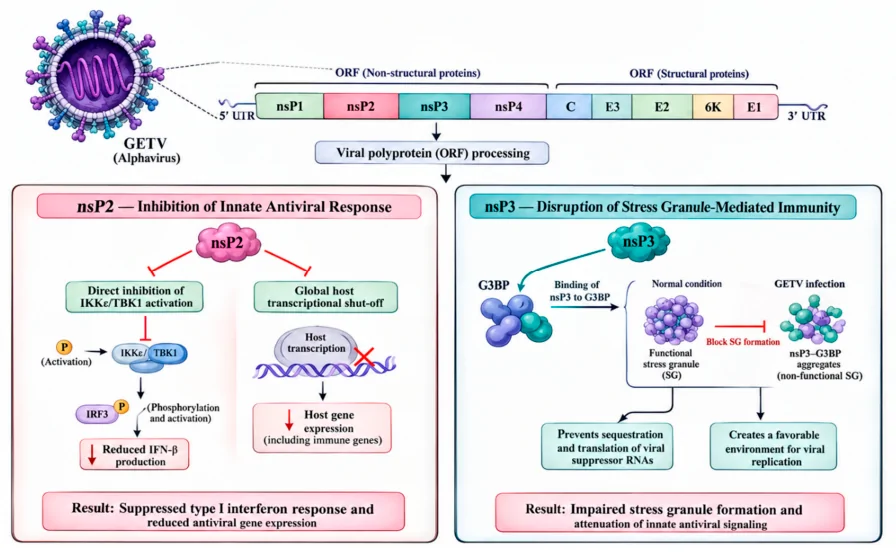
**Molecular mechanisms of GETV immune evasion mediated by nsP2 and nsP3.** GETV nsP2 suppresses type I interferon responses via inhibition of IKKε/TBK1 pathways and global host transcriptional shut-off [45], whereas nsP3 interacts with G3BP and disrupts functional stress granule formation [36]. Collectively, these molecular pathways bypass or reduce innate antiviral responses and create a cellular environment supportive to viral replication. The figure was designed using Adobe Photoshop 2020.

GETV nsP2 may potentially disrupt the antiviral signaling that occurs after IFN. Studies have indicated that nsP2 obstructs the phosphorylation of STAT1 and its nuclear translocation, leading to compromised JAK–STAT signaling and diminished expression of interferon-stimulated genes (ISGs) [26]. Consequently, nsP2 may impair two separate phases of the type I IFN response: the synthesis of IFN by hindering IRF3 activation [45] and the cellular reaction to IFN by obstructing STAT1-dependent signaling [26] (Figure 4). The C-terminal amino acids K648 and R649 of nsP2 have a role in its nuclear localization and are linked to its ability to counteract interferon and its pathogenicity in murine models [26]. These results indicate a functional relationship between nsP2-facilitated immune evasion and viral pathogenicity.

Recent findings have suggested that GETV nsP2 may furthermore interact with IKKε, polyubiquitination, and phosphorylation associated with RIG-I-like receptor signaling (Figure 4), therefore offering another method for inhibiting IFN-β and ISG responses [34,45]. Collectively, these results suggest that nsP2 is capable of influencing several regulatory checkpoints in innate antiviral signaling instead of acting via one specific mechanism. Nonetheless, the exact impact of each pathway on tissue-specific pathology has yet to be identified. The question of whether these immune-evasion mechanisms function concurrently during GETV infection, and how their respective impacts differ between host species and tissues, remains a significant unsolved question.

In addition to nsP2-induced interferon antagonism, nsP3 can also manipulate cellular stress response in GETV infection. GETV infection stimulates the PKR/eIF2α signaling cascade and prompts the formation of cytoplasmic aggregates containing G3BP (Figure 4). Nevertheless, these formations are distinct from authentic stress granules. The hypervariable domain (HVD) of nsP3 interacts with the NTF2 domain of G3BP1 and disrupts the assembly of authentic stress granules [36]. More broadly, interactions between alphavirus non-structural proteins and host RNA-binding proteins can modify cellular stress responses and influence viral replication [54]. However, the extent to which these broader mechanisms apply to GETV requires further investigation.

Together, nsP2-mediated interferon antagonism and nsP3-mediated modulation of stress granule formation may facilitate GETV replication; however, their contributions to tissue dissemination and host-specific pathology remain unclear.

### 5.3. Cytokine and Inflammatory Responses

Innate immune responses, such as inflammation, help in the prevention of viral infection, but GETV triggers sustained production of inflammatory mediators, such as interleukin-6 (IL-6), tumor necrosis factor α (TNF-α) and interferon γ (IFN-γ) [19,53]. These mediators stimulate host immune response, but excessive or dysregulated inflammation may also trigger deeper tissue damage. The clinical and pathological outcomes of GETV infection vary among host species, with reproductive disorders and increased mortality reported in pigs, while fever, rash, and limb edema are obvious in horses [38,55].

However, whether this difference in viral pathology is influenced by variations in cytokine responses, viral replication, or tissue-specific susceptibility remains unclear. Although GETV nsP2 can suppress type I interferon responses [45], the relationship between interferon antagonism, inflammatory signaling, and tissue damage has not been fully established. Further studies are therefore needed to determine how cytokine responses contribute to GETV-associated pathology in different hosts.

## 6. Tissue Tropism and Pathogenesis Across Various Hosts

**Figure 5:**
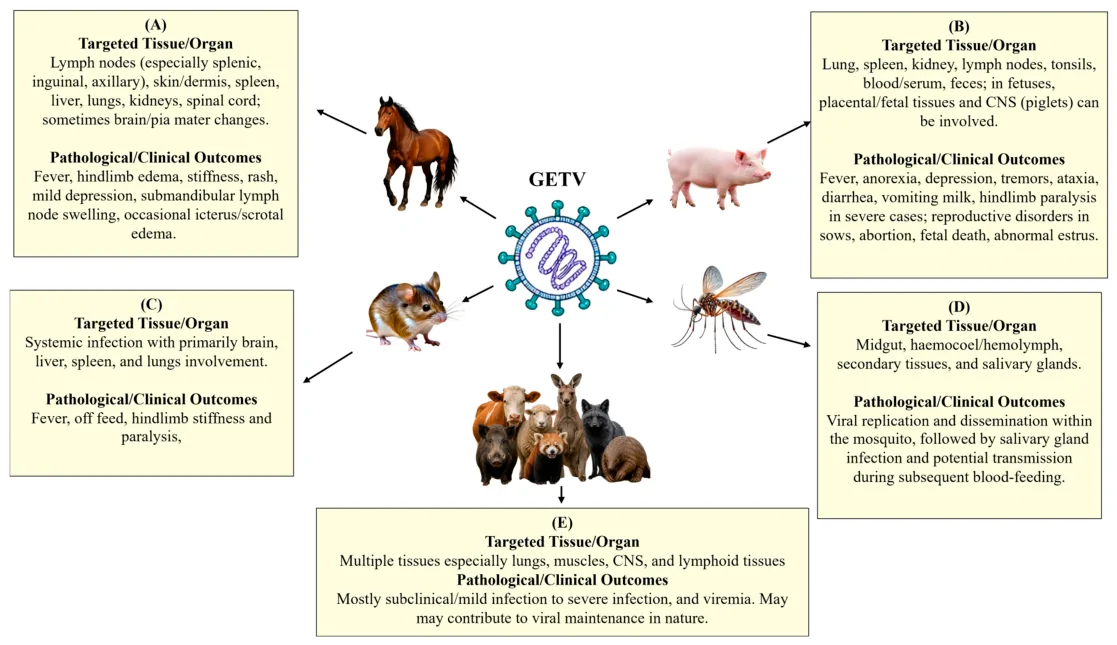
**Host-specific tissue tropism and pathological outcomes of GETV infection.** Summary of reported clinical outcomes and tissue-associated findings in major host species and experimental models. (**A**) In horses, GETV infection is associated with systemic disease characterized by fever, rash, edema, and musculoskeletal manifestations, with viral RNA detected in blood during natural infection [6,56]. (**B**) In pigs, GETV infection is associated with systemic viral dissemination, reproductive disorders, and neonatal disease; viral loads have been detected in blood and multiple tissues, while histopathological lesions have been reported in affected organs [32,58]. (**C**) Experimental mouse models develop systemic infections, with predominant involvement of the brain, liver, spleen, and lungs [19]. (**D**) In mosquito vectors, viral replication typically begins in the midgut, followed by dissemination through the hemocoel to secondary tissues and the salivary glands, facilitating transmission during subsequent blood feeding [63,64]. (**E**) Other hosts, including cattle, sheep, wild boar, kangaroos, red pandas, blue foxes, and pangolins, generally exhibit mild/subclinical-to-severe infections and may contribute to viral maintenance in nature [15,38,60].

Despite the conserved replication machinery, GETV gives distinct pathological and clinical outcomes among various hosts (Figure 5), which explain tissue tropism and disease severity as the combined effects of different factors, such as cellular permissiveness, viral entry, replication efficiency, and host immune responses. Thus, host-specific pathology represents the outcome of interactions between viral properties and the biological characteristics of the infected host.

### 6.1. Horses

Horses are an important host in GETV outbreaks and commonly develop acute febrile illness, limb edema, urticarial rash, and lymphadenopathy [2]. The evidence for infection in naturally affected horses includes detection of GETV RNA in blood samples, as demonstrated in a naturally infected racehorse in China [56]. Experimental and epidemiological studies also provide evidence of GETV replication and host cell permissiveness in different cellular systems. Serological studies provide additional evidence of previous exposure to horse populations, but do not by themselves demonstrate active tissue infection [57].

The distribution of GETV within specific equine tissues and the mechanisms responsible for tissue-specific pathology remain incompletely defined. Although lymphoid and vascular tissues have been proposed as contributing to viral dissemination and the development of edema, direct evidence establishing endothelial dysfunction as a primary molecular mechanism of GETV pathogenesis in horses remains limited. Therefore, vascular leakage and inflammatory changes should be considered possible contributors to the observed edema and cutaneous lesions rather than established mechanisms. Overall, the available evidence supports systemic infection in horses, but remains insufficient to define a specific cellular or tissue-level mechanism underlying equine tissue tropism.

### 6.2. Pigs

In pigs, GETV infection has been associated with reproductive disorders, including abortion, fetal mummification, stillbirth, and neonatal mortality [32]. These observations are supported primarily by clinical and reproductive outcomes documented during natural and experimental infections. GETV has also been isolated from porcine clinical samples, providing direct evidence of productive viral infection in pigs [32,58]. In experimental infections, viral loads have been detected in blood and multiple tissues, demonstrating systemic dissemination beyond the initial site of infection [7,11,58].

Evidence for involvement of fetal and reproductive tissues should, however, be distinguished from evidence of a specific mechanism of reproductive tropism. Detection of viral material in a tissue can indicate exposure or viral presence, but does not necessarily demonstrate sustained productive replication unless supported by virus isolation, increasing viral titers, antigen detection, or other direct measures of replication. Similarly, reproductive failure and fetal or neonatal abnormalities establish an association between GETV infection and reproductive disease but do not, by themselves, establish placental permissiveness or a specific mechanism of transplacental transmission.

The maternal–fetal interface and developmental stage of the fetus may contribute to the outcome of GETV infection, but these mechanisms remain insufficiently characterized. Therefore, the available evidence supports systemic infection and an association between GETV infection and reproductive disease in pigs, whereas the precise cellular and tissue mechanisms responsible for fetal infection, placental involvement, and reproductive pathology require further experimental investigation.

### 6.3. Tissue Tropism in Experimental Rodent Models

Rodents, such as mice, are the most commonly used experimental models to study GETV virulence and dissemination. Experimental infections of different mice have shown viral dissemination in various organs, including the brain, liver, muscles, and joints. Studies have revealed that tissue tropism depends on the route of administration, highlighting the infection route as an important landmark to interpret tissue tropism [19]. Recent studies have suggested AG129 mice as an ideal model to understand generalized GETV infections, virulence, and systemic viral lesions; however, the lack of interferon type I and II makes them difficult to compare with immunocompetent hosts [59].

### 6.4. Other Hosts

GETV shows variable clinical and pathological outcomes in hosts other than horses and pigs. Viral RNA has been detected and infectious virus isolated from cattle with clinical signs, including fever, but with limited clinical signs in some seropositive animals [8]. In blue foxes, upon GETV infection, fever, anorexia, depression, and sometimes neurological signs have been observed, with mild neuronal degeneration and vascular inflammatory infiltration in the brain and severe hemorrhagic pneumonia in fatal cases [9]. These results indicate that GETV-induced disease in this host might occur in both neurological and respiratory tissues.

By contrast, the identification of a Group IV GETV strain in Chinese pangolins broadened the known host spectrum, but did not provide information about the severity of the disease or tissue tropism in this host [60]. The antibodies of GETV have also been found in other domestic and wild animals, such as sheep [61] and wild boars [11], but the detection of these antibodies cannot confirm the presence of an active infection or pathogenicity [2]. Reports regarding kangaroos should be viewed with some caution, as the earlier Australian GETV serological reports may have been affected by a cross-reaction with the Ross River virus [62]. These findings suggest that GETV infection can have different outcomes across hosts; however, the roles of different host species as amplifying hosts, reservoirs, or incidental hosts remain unclear. Comparative studies are needed to establish host-specific tissue tropism, elucidate the mechanisms underlying pathogenesis, and clarify the contributions of different host species to GETV maintenance and transmission.

Thus, host-specific tissue tropism in GETV infection may be determined by replication competence, cellular permissiveness, and host immune evasion. Therefore, differences in such factors could transform a self-limiting disease into systemic dissemination with severe tissue tropism. To understand this GETV pathogenesis, researchers should focus on defining the factors that determine productive viral replication within individual tissues.

## 7. Vector–Host–Virus Interactions

### 7.1. Transmission Dynamics

**Figure 6:**
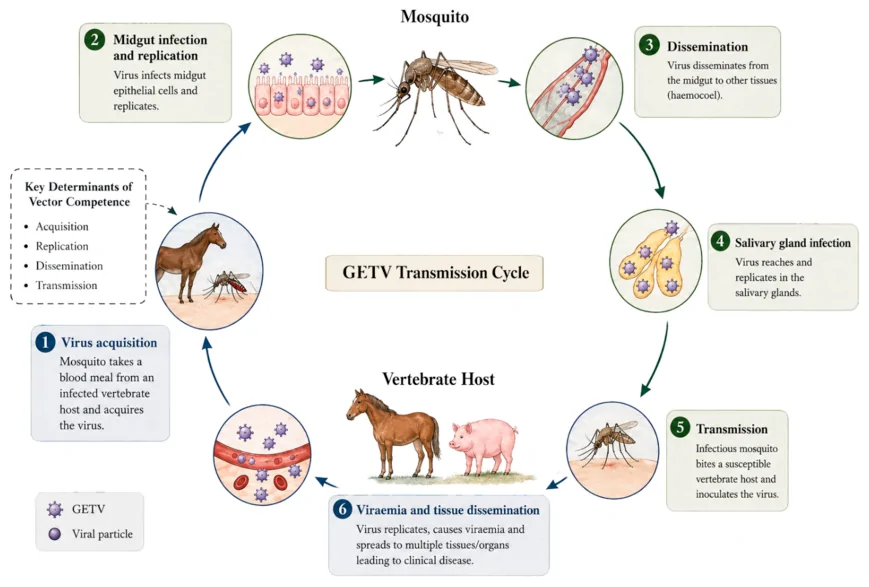
**Transmission cycle of GETV between mosquito vectors and vertebrate hosts.** The cycle comprises virus acquisition from a viremic vertebrate host, replication in the mosquito midgut, dissemination to secondary tissues and salivary glands, and transmission to a susceptible vertebrate host during subsequent blood feeding. Following infection, GETV can produce viremia and disseminate to multiple tissues, contributing to host-specific clinical outcomes. The figure was designed using Adobe Photoshop 2020.

GETV has a complex transmission cycle, including mosquito vectors and vertebrate hosts as well as viral genetic factors that potentially influence replication and fitness in both hosts and environments. This dynamic interplay determines circulating strains and their geographical distribution, along with amplifying host species [13,24]. Mosquitoes acquire viruses during blood feeding, support viral replication, and subsequently transmit infectious viruses to susceptible vertebrate hosts (Figure 6). These stages put selective pressure on GETV, supporting viral variants that can maintain sufficient replication and transmission efficiency across both hosts and environments.

### 7.2. Mosquito Vector

Mosquitoes are the primary vectors for GETV spread. Several experimental and field studies indicate that Culex is a main genus involved in natural transmission cycles. Among these, *Culex tritaeniorhynchus* is considered the principal vector, especially in agricultural or peri-urban areas in East and Southeast Asia, where they frequently interact with amplifying vertebrate hosts, including pigs and horses. In addition to *Culex tritaeniorhynchus*, other species including *Culex pipiens*, *Culex bitaeniorhynchus*, and certain *Aedes* mosquitoes have also been found to be involved in the transmission of GETV, but their relative contribution varies according to the geographical and ecological conditions [63,64].

### 7.3. Vector–Host Interface in Tropism Determination

Although mosquitoes are the main vectors for GETV transmission, limited information is available on its viral evolution and adaptation within mosquito hosts. However, recent studies have revealed that GETV replicates efficiently in certain (*Culex and Aedes*) mosquito-derived cell lines, leading to different viral titers and cytopathic effects, suggesting differences in vector cell permissiveness [48,63]. As already described, GETV utilizes heparan sulfate as an attachment factor in mammalian cells; however, it is not clear whether the same entry determinants are useful in mosquitoes or not.

Moreover, the composition of glycosaminoglycans, endocytic routes, and membrane remodeling is significantly different in mosquito cells and may influence E1/E2-mediated entry and fusion. Recent experimental evidence further indicates that E1/E2 glycosylation can differentially influence GETV fitness in mammalian and mosquito systems, highlighting envelope glycosylation as a potential determinant of cross-host adaptation [21]. Furthermore, GETV must overcome mosquito midgut infection and escape barriers to disseminate into secondary tissues, including salivary glands, to enable transmission. The efficiency of these processes varies among mosquito species, highlighting that vector competence may influence GETV transmission dynamics [63]. Genetic variations in the E1/E2 envelope glycoproteins and changes in their glycosylation patterns may also affect viral fitness and host interactions. Recent GETV-specific studies have demonstrated that envelope glycosylation influences mosquito replication, receptor interactions, and virulence in mammalian models [21,50]. However, whether these changes represent adaptations specifically driven by tissue-specific infection during transmission between particular hosts and vectors remains to be established.

The current evidence indicates that GETV pathogenesis involves multiple viral and host factors, although their experimental support varies. As already described, GETV-specific studies have established roles for envelope-mediated attachment, nsP2-dependent interferon antagonism, and nsP3-G3BP interactions. However, the mechanisms governing tissue-specific permissiveness, host-dependent pathology, and vector–host adaptation remain incompletely understood. Table 3 distinguishes the experimentally demonstrated GETV mechanisms from the findings inferred from related alphaviruses.

**Table 3:** Evidence synthesis of molecular and host determinants of GETV pathogenesis.

Pathogenic Component	GETV-Specific Evidence	Inferred Evidence
Viral entry and attachment	HS-mediated attachment; E2-K253R effects; GETV-specific LDLR/entry factor studies [21,22,39,42].	General E1-mediated fusion mechanisms were not directly tested in GETV [41].
Intracellular replication	GETV nsP3–G3BP interaction [36]; GETV capsid–RNA interaction and 6K studies [28,29].	Detailed nsP1 capping, nsP4 polymerase, and replication complex mechanisms [33,37].
Immune evasion	GETV nsP2-mediated IFN antagonism [26,34,45]; nsP3–G3BP effects [36].	Broader alphavirus immune evasion mechanisms and canonical signaling details [51,52].
Inflammation and tissue tropism	GETV-associated lesions and host-dependent pathology in animal studies [19,38,58].	Proposed endothelial/inflammatory mechanisms derived from other arthritogenic alphaviruses [51,52,65].
Viral genetic variation	GETV E2-K253R and envelope glycosylation studies [21,39,50]; isolate-specific replication differences [48].	Mutation-driven tropism and adaptation mechanisms were established in CHIKV, SINV or RRV [41,66].
Vector–host adaptation	GETV mosquito vector-competence studies [63,64]; envelope glycosylation effects across mosquito and mammalian systems [21,50].	General alphavirus mechanisms of cross-host adaptation [41,51,66].

**Note:** “GETV-specific evidence” refers to findings directly demonstrated using GETV infection, viral proteins, mutants, or GETV-infected experimental systems. “Inferred evidence” refers to mechanisms supported primarily by studies of related alphaviruses or by associations that have not been functionally validated in GETV. Inferred evidence is included to provide mechanistic context but is not considered proof of an equivalent mechanism in GETV. The evidence classification reflects the current state of the literature and highlights areas requiring further experimental validation.

## 8. Genetic Diversity of GETV in Pathogenesis and Host Adaptation

### 8.1. Genetic Diversity and Phylogenetic Structure

**Figure 7:**
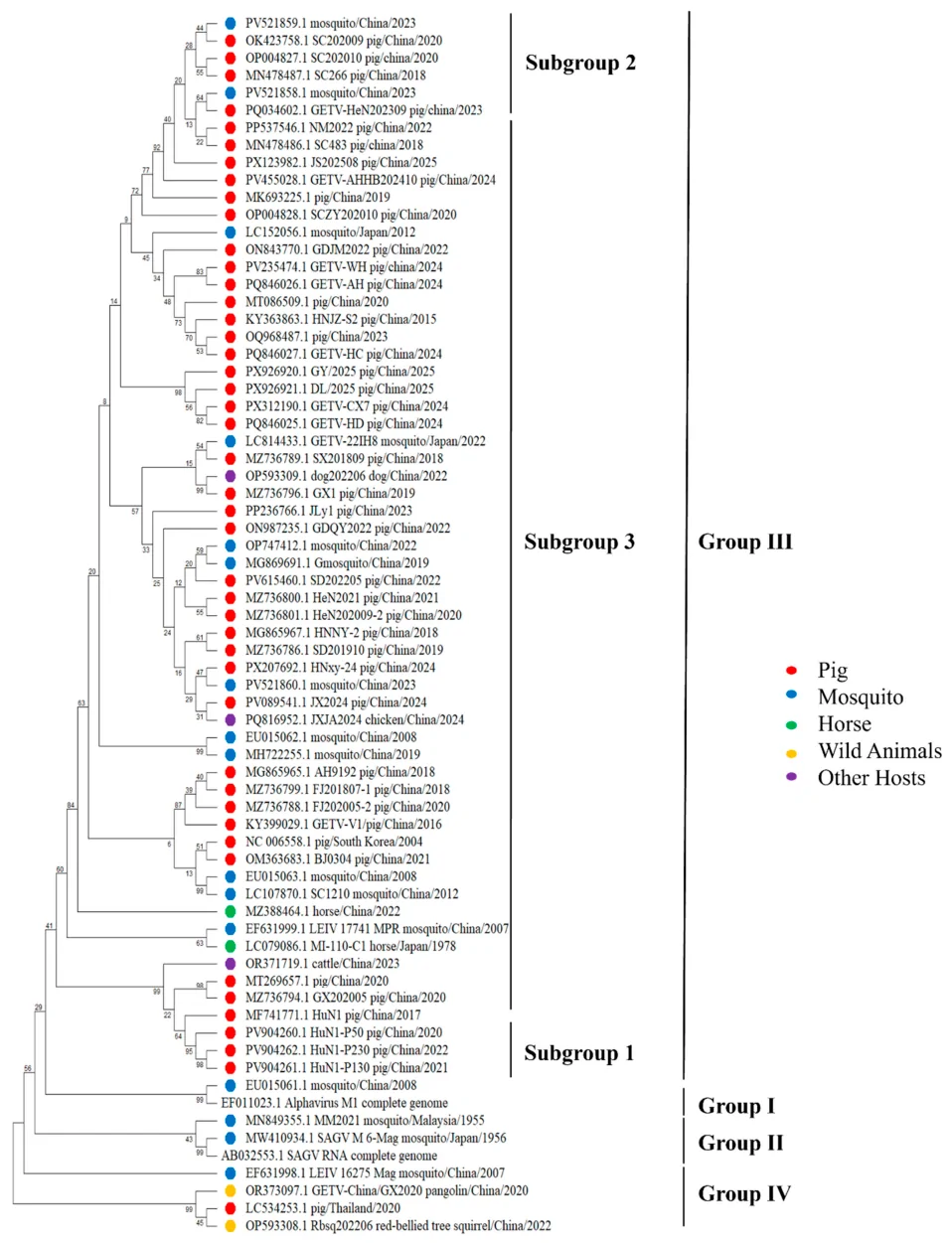
**Maximum-likelihood phylogenetic analysis of GETV E2 gene sequences in the context of related alphaviruses.** GETV E2 nucleotide sequences were retrieved from the NCBI GenBank database, together with selected related alphavirus sequences to provide comparative phylogenetic context. The GETV sequences represent different host species, geographic regions, collection years, and major genetic groups. To reduce redundancy, when closely related sequences were available from the same or similar geographic regions and were reported in related studies, representative sequences were retained. Multiple-sequence alignment was performed using MUSCLE implemented in MEGA12, followed by manual inspection. The final alignment comprised 1152 nucleotide positions, with partial deletion applied using a minimum site-coverage threshold of 95%. The maximum-likelihood tree was inferred using the Tamura–Nei model with a proportion of invariant sites (TN93 + I), selected based on the Bayesian Information Criterion (BIC), with 1000 bootstrap replicates to assess branch support. Bootstrap values are shown at the relevant nodes. The GETV sequences are distributed across the major genetic groups, while selected related alphavirus sequences provide comparative phylogenetic context. Host species are indicated by different colors. Accession numbers and available information on host, collection date or year, isolation source, and geographic origin are provided in Supplementary Table S1. The tree illustrates genetic relationships among the sampled sequences; phylogenetic clustering alone does not establish host adaptation, lineage-specific ecological niche partitioning, transmission direction, or differences in pathogenicity.

Recent genomic surveillance studies have indicated the ongoing genetic diversification of GETV, contributing to the genetic diversity observed among circulating strains [67]. Phylogenetic analyses have classified GETV sequences into four genetic groups (Groups I–IV), with Group III showing substantial diversification and including strains associated with recent outbreaks [13]. Group IV includes sequences detected in wildlife, including Chinese pangolins (Figure 7) [60]. These findings demonstrate the genetic diversity and broad host range represented among sampled GETV sequences. However, phylogenetic clustering and host-associated detection alone do not establish host adaptation, ecological niche partitioning, or differences in pathogenicity; these relationships require additional epidemiological and functional evidence.

**Figure 8:**
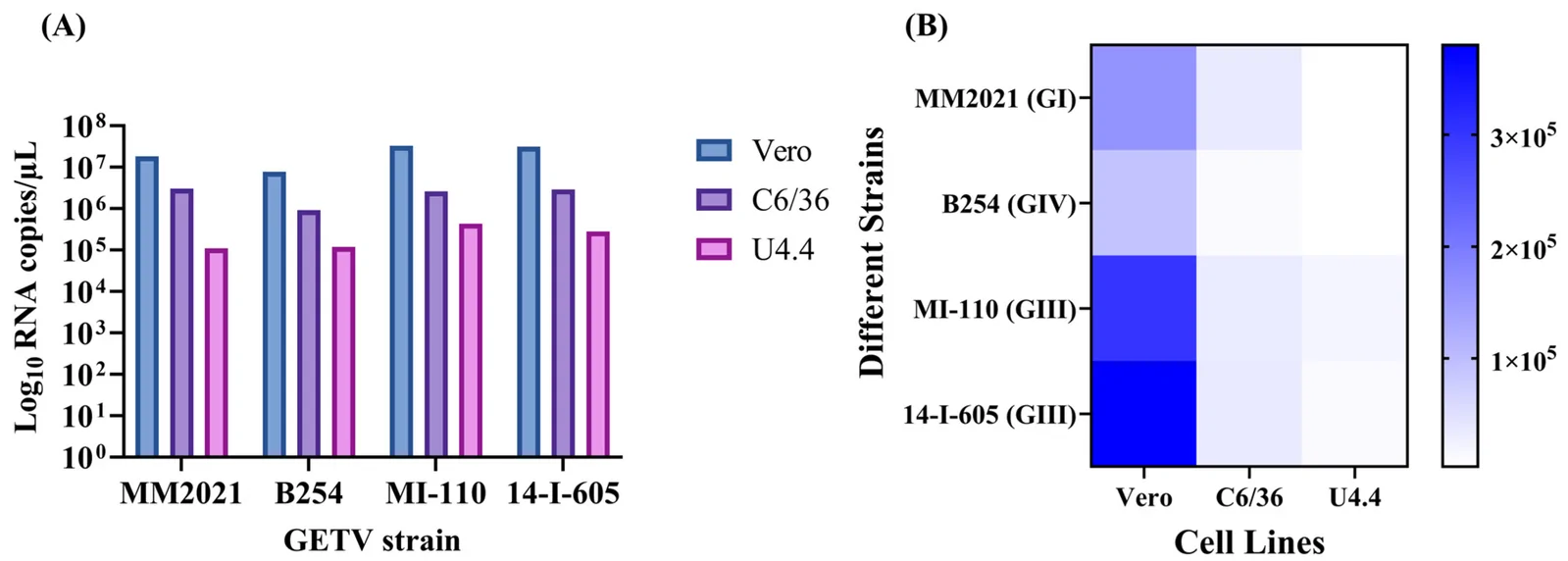
**Differential replication phenotypes of GETV isolates across various cell lines.** (**A**) Bar graphs represent the growth of GETV in terms of the mean viral RNA copies across four GETV strains GI (MM2021), GIV (B254), and GIII (MI-110 and 14-I-605) in Vero, C6/36 and U4.4 cells. (**B**) Heatmap summarizing the corresponding replication phenotypes across the three cell lines. GIII isolates, particularly 14-I-605 and MI-110, exhibit higher replication than the GI and GIV isolates. The contrasting patterns between mammalian and mosquito-derived cells highlight the influence of the cellular environment on GETV replication and provide experimental evidence for genotype-dependent differences in viral fitness. Adapted and replotted from Mohamed-Romai-Noor et al. (2022) [48] using GraphPad Prism 10.5.0 version, licensed under the Creative Commons Attribution (CC BY) license.

Group I and II are considered geographically restricted groups with limited outbreaks; however, Group III is associated with more as well as larger outbreaks in Asia [2,4]. Comparative experimental studies have also identified differences in replication among GETV isolates in different cell systems. For example, Mohamed-Romai-Noor et al. compared MM2021 (GI), MI-110 and 14-I-605 (GIII), and B254 (GIV) [48]. The GIII isolates, particularly 14-I-605, showed higher replication in the tested cell system than GI and GIV (Figure 8). These findings indicate isolate-dependent differences in replication under the experimental conditions used. However, higher replication in cultured cells does not necessarily indicate greater pathogenicity in animals or increased epidemic potential.

These outcomes suggest that epidemic behavior is not the reflection of a single genetic determinant, but reflects an interaction between host cell permissiveness and viral strains. However, higher replication in a specific cell system should not be considered as enhanced pathogenicity in an animal experiment, as the host immune system and cellular adaptation may impose a different selection pressure.

### 8.2. Viral Mutations and Phenotypic Consequences

Genetic diversity in GETV reflects the accumulation of sequence variations across viral lineages. Comparative genomic studies have identified genetic differences among circulating strains, including those associated with recent outbreaks [13,67]. However, the extent to which these differences determine host adaptation, transmission efficiency, or pathogenicity remains incompletely understood. Phylogenetic clustering reveals genetic relationships and the distribution of sampled lineages across hosts and geographic regions, but does not independently establish the mechanism governing viral diversification. Environmental conditions and vector ecology may influence GETV transmission and dispersal, although their direct contribution to genetic evolution requires further investigation.

## 9. Experimental Modeling for GETV Pathogenesis

Experimental modeling is essential for linking viral genetic variation with replication, tissue tropism, immune responses, and pathogenesis in GETV infection. Each experimental system recapitulates distinct aspects of GETV pathogenesis and host adaptation.

### 9.1. Cell Culture Models and Their Functional Role

Cell culture systems are important pre-clinical tools to study viral behavior, including replication, cytopathic effects, molecular mechanisms, and virus–host interactions. BHK-21 and Vero are the most commonly used mammalian cell lines to study viral propagation, reverse genetics, and replication, while PK-15 provides pig-associated cellular context to investigate host-specific viral phenotypes [48,67,68]. Mouse Leydig (TM3) cells provide an example to evaluate ROS-mediated autophagy during GETV infection [69]. Furthermore, mosquito-derived C6/36 and U4.4 cells are also important to understand vector-associated replication and differences in cellular permissiveness [48]. Cell culture studies reveal host-dependent differences in GETV replication, but cannot fully reproduce in vivo pathogenesis due to the absence of tissue architecture, multicellular interactions, and systemic immune responses.

### 9.2. Animal Models

Animal models are essential to explore the physiological relevance of viral dissemination, role of viral determinants, host responses, and pathogenesis. BALB/c and ICR mice are well-established models to study GETV pathogenesis and viral mutations [19,21,48]. As described earlier, an AG129 mouse, a new type I and type II interferon receptor-deficient, demonstrated severe GETV infection with symptoms including fetal systemic infection and a higher viral load, along with lesions across multiple organs. Even though this model does not have interferon signaling compared to immunocompetent hosts, it represents a sensitive system to study virulence and vaccine [59].

Moreover, pig models, including piglets and pregnant sows, provide an ideal experimental model to study GETV infections, as they can provide an estimation of a real-time outbreak [38]. Their use is particularly valuable for investigating age- and physiological-state-dependent pathogenesis that cannot be adequately studied in mice.

### 9.3. Limitations and Complementarity

Each experimental system helps to study one aspect of GETV infection. Cell lines are valuable for mechanistic studies but cannot reproduce tissue-level interactions, while mouse models provide controlled analysis of dissemination and pathology but do not fully reproduce natural disease in horses or pigs. Mosquito-derived cell lines likewise cannot substitute for whole-vector competence. Therefore, integrating cellular, vector, and animal models remains essential for linking molecular mechanisms with host-specific GETV pathogenesis.

## 10. Prevention and Control Strategies

GETV pathophysiology is better understood, but no antiviral medication is available for regular infection control. This gap is significant because serious illness in newborn pigs and reproductive losses in breeding populations require an effective and scalable swine vaccine [2,38]. Since the first Japanese epidemic, horses have received an inactivated vaccination. Equine immunization is available [57], whereas pigs have no corresponding approved vaccine, demonstrating host species’ different disease-control techniques.

Emerging vaccine platforms, including self-amplifying RNA and viral vector-based vaccines, may offer improved immunogenicity and rapid adaptability against evolving GETV strains. The discovery of conserved and functionally significant areas in E1/E2 may promote rational antigen selection, but broad protective vaccinations should account for antigenic heterogeneity among circulating lineages [21]. Although there is no antiviral treatment against GETV, researchers are using modified treatments already established against other alphaviruses; however, their efficacy remains uncertain [38]. Furthermore, understanding of nsP2- and nsP3-associated host immune evasion mechanisms may help in developing antiviral treatments against GETV, as described in other alphaviruses [44,70].

From an epidemiological point of view, surveillance and detection are considered basic parameters to correlate pathogenesis to epidemic field outbreaks. GETV diagnosis in livestock and wildlife relies on PCR for RNA detection in blood, tissues, and mosquitoes, alongside serological assays like ELISA [61,67]. Surveillance data can help identify novel host–vector combinations, outbreak hotspots, and populations at risk, while genomic integration with clinical data and tissue scoring can track lineages with expanding host tropism or increasing virulence [61].

Another possible control strategy is vector control, especially in areas with higher risks. Several approaches have been used, such as integrated vector surveillance, larval source reduction, adult mosquito control, and other biosecurity measures [13]. Together, these approaches may help to translate understanding of GETV transmission dynamics along with pathogenesis into practical solutions to develop control strategies.

## 11. Knowledge Gaps and Future Research Directions

Despite substantial progress in understanding GETV molecular biology, transmission, and pathogenesis, several important questions remain unresolved. A fundamental unresolved question concerns the relative contributions of LDLR and other cellular factors to productive GETV entry, tissue tropism, and host specificity, which remain incompletely defined [42].

A second major challenge is determining how viral and host factors interact to produce distinct tissue tropism and disease phenotypes across species. Comparative studies of E1/E2 variation, glycosylation, non-structural proteins, and host antiviral responses, combined with single-cell and spatial approaches, could help identify the cellular determinants underlying host- and tissue-specific susceptibility [19,48,50].

Functional validation of candidate viral determinants is also needed. Reverse genetics studies targeting naturally occurring amino acid substitutions and glycosylation sites could directly link viral genotypes with replication, virulence, tissue distribution and vector competence, moving beyond the associations identified through comparative genomics.

Finally, greater integration of molecular, ecological and epidemiological data is required to understand cross-host adaptation and predict the emergence of potentially important GETV lineages. Combining genomic surveillance with mosquito, livestock and wildlife monitoring within a One Health framework could improve lineage tracking, outbreak prediction and the identification of emerging host-adaptation patterns.

## 12. Conclusions

**Figure 9:**
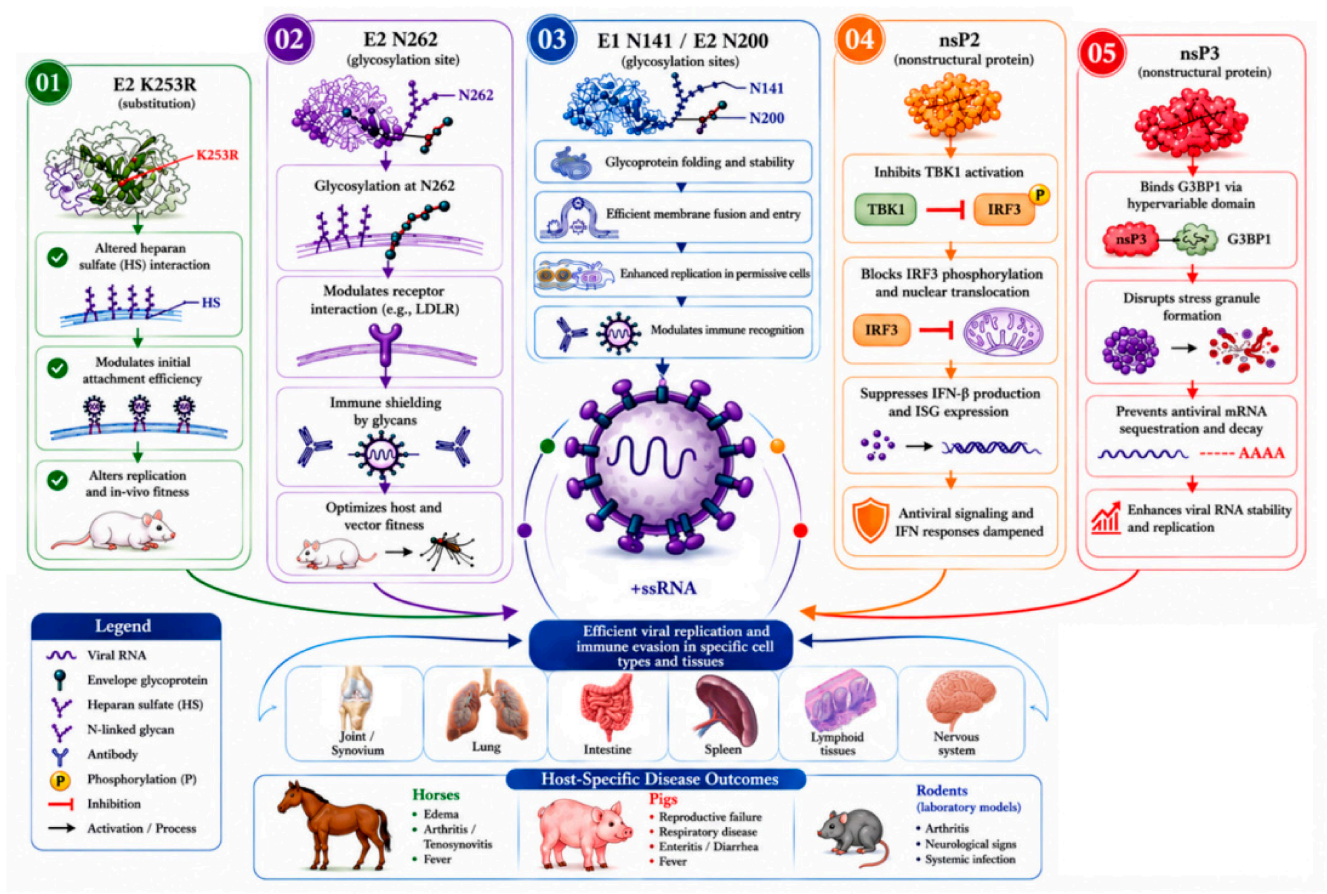
**Integrated molecular and host determinants of GETV pathogenesis and host-specific disease.** Viral structural and non-structural proteins contribute to distinct stages of GETV infection, including cellular attachment, entry, replication, and evasion of innate antiviral responses. E2 K253 and glycosylation-associated residues influence host cell interaction and viral fitness, whereas nsP2 and nsP3 modulate antiviral signaling and cellular stress responses. These molecular effects contribute to tissue-specific viral replication and, together with host and vector factors, shape distinct disease outcomes across horses, pigs and rodent models. The proposed links between individual molecular mechanisms and broader host- or tissue-specific disease outcomes represent an integrated interpretation of the available evidence and should not necessarily be regarded as direct causal relationships.

GETV pathogenesis is determined by a dynamic interaction between viral genetic variation, cellular entry and replication, host antiviral responses, tissue-specific permissiveness and vector–host adaptation. E1/E2 glycoproteins, particularly their receptor-interacting and glycosylated regions, contribute to viral entry and host adaptation, while non-structural proteins regulate replication and antagonize antiviral responses. Together, these mechanisms help explain the diverse tissue distribution and disease phenotypes observed across GETV-infected hosts (Figure 9).

The available evidence indicates that GETV tissue tropism cannot be attributed to a single viral determinant. Instead, it emerges from the combined effects of viral entry, intracellular replication, immune evasion, host physiology, and evolutionary pressures operating across mosquito and vertebrate hosts. Therefore, reverse genetics, functional virology, advanced cellular models, and genomic surveillance must be integrated to define host-specific pathogenesis and inform vaccine, therapeutic, and surveillance development.

## Data Availability

The nucleotide sequences analyzed in the phylogenetic analysis (Figure 7) were obtained from the NCBI GenBank database. The corresponding accession numbers and sequence metadata are provided in Supplementary Table S1.

## Supplementary Materials

The following supporting information can be downloaded at: https://www.mdpi.com/article/10.3390/vetsci13101092/s1, Table S1.

## Abbreviations

The following abbreviations are used in this manuscript:

GETV	Getah virus
E1	Envelope glycoprotein 1
E2	Envelope glycoprotein 2
nsP1	Non-structural protein 1
nsP2	Non-structural protein 2
nsP3	Non-structural protein 3
nsP4	Non-structural protein 4
IFN	Interferon
IFN-β	Interferon beta
IFN-γ	Interferon gamma
ISGs	Interferon-stimulated genes
JAK–STAT	Janus kinase–signal transducer and activator of transcription
TBK1	TANK-binding kinase 1
IRF3	Interferon regulatory factor 3
RIG-I	Retinoic acid-inducible gene I
MDA5	Melanoma differentiation-associated protein 5
MAVS	Mitochondrial antiviral-signaling protein
PKR	Protein kinase R
G3BP1	Ras GTPase-activating protein-binding protein 1
AG129	Mice deficient in type I and type II interferon receptors
CHIKV	Chikungunya virus
SINV	Sindbis virus

## References

[1] Zhang H.-L., Hu T.-S., Zhang F.-Q. (2020). Progress on Getah virus research in China and its public health significance. Chin. J. Zoonoses.

[2] Li B., Wang H., Liang G. (2022). Getah virus (Alphavirus): An emerging, spreading zoonotic virus. Pathogens.

[3] Li Y.-Y., Liu H., Fu S.-H., Li X.-L., Guo X.-F., Li M.-H., Feng Y., Chen W.-X., Wang L.-H., Lei W.-W. (2017). From discovery to spread: The evolution and phylogeny of Getah virus. Infect. Genet. Evol..

[4] Irekeola A.A., Shueb R.H. (2025). Global seroprevalence and distribution of Getah virus in domestic and wild animals: A systematic review and meta-analysis. Vet. World.

[5] Gromashevski’i V., Aristova V., Morozova T., Skvortsova T., Gushchina E., Petrova E., L’vov D. (2000). Isolation of Getah virus (Togaviridae, Alfavirus) strains in North-Eastern Asia. Vopr. Virusol..

[6] Bannai H., Ochi A., Nemoto M., Tsujimura K., Yamanaka T., Kondo T. (2016). A 2015 outbreak of Getah virus infection occurring among Japanese racehorses sequentially to an outbreak in 2014 at the same site. BMC Vet. Res..

[7] Yang T., Li R., Hu Y., Yang L., Zhao D., Du L., Li J., Ge M., Yu X. (2018). An outbreak of Getah virus infection among pigs in China, 2017. Transbound. Emerg. Dis..

[8] Liu H., Zhang X., Li L.-X., Shi N., Sun X.-T., Liu Q., Jin N.-Y., Si X.-K. (2019). First isolation and characterization of Getah virus from cattle in northeastern China. BMC Vet. Res..

[9] Shi N., Li L.-X., Lu R.-G., Yan X.-J., Liu H. (2019). Highly pathogenic swine Getah virus in blue foxes, eastern China, 2017. Emerg. Infect. Dis..

[10] Zhao M., Yue C., Yang Z., Li Y., Zhang D., Zhang J., Yang S., Shen Q., Su X., Qi D. (2022). Viral metagenomics unveiled extensive communications of viruses within giant pandas and their associated organisms in the same ecosystem. Sci. Total Environ..

[11] Huang J., Song D., Wei J., Xie B., Yang Q., Xu J., Zhang F. (2025). First report on identification and genetic characterization of Getah virus in wild boar in China. Front. Microbiol..

[12] Wang A., Zhou F., Liu C., Gao D., Qi R., Yin Y., Liu S., Gao Y., Fu L., Xia Y. (2022). Structure of infective Getah virus at 2.8 Å resolution determined by cryo-electron microscopy. Cell Discov..

[13] Yuan Y., Hao Y., Peng C., Zhang D., Ma W., Xiao P., Li N. (2025). From transmission to adaptive evolution: Genomic surveillance of Getah virus. Front. Cell. Infect. Microbiol..

[14] Nemoto M., Bannai H., Tsujimura K., Kobayashi M., Kikuchi T., Yamanaka T., Kondo T. (2015). Getah virus infection among racehorses, Japan, 2014. Emerg. Infect. Dis..

[15] Ren T., Mo Q., Wang Y., Wang H., Nong Z., Wang J., Niu C., Liu C., Chen Y., Ouyang K. (2020). Emergence and phylogenetic analysis of a Getah virus isolated in southern China. Front. Vet. Sci..

[16] Fukunaga Y., Kumanomido T., Kamada M. (2000). Getah virus as an equine pathogen. Vet. Clin. N. Am. Equine Pract..

[17] Yuen N.K. (2026). Getah virus, a re-emerging arbovirus: Epidemiology, pathogenesis, diagnostics, and biosecurity. Virology.

[18] Zhang Y., Yu J., Tan L., Wang X., Li R., Kim D.Y. (2021). Complete genetic dissection and cell type-specific replication of old world alphaviruses, getah virus (GETV) and sagiyama virus (SAGV). J. Microbiol..

[19] Jian Z., Jiang C., Zhu L., Li F., Deng L., Ai Y., Lai S., Xu Z. (2024). Infectivity and pathogenesis characterization of getah virus (GETV) strain via different inoculation routes in mice. Heliyon.

[20] Lan J., Fang M., Duan L., Liu Z., Wang G., Wu Q., Fan K., Huang D., Ye Y., Wan G. (2024). Novel porcine getah virus from diarrheal piglets in Jiangxi province, China: Prevalence, genome sequence, and pathogenicity. Animals.

[21] Shi N., Qiu X., Tian J., Cao X., Zhu X., Liu B., Zhang X., Zhang H., Wang H., Lu H. (2026). Glycosylation of GETV E2 promotes pathogenesis in animal models. Proc. Natl. Acad. Sci. USA.

[22] Qiu X., Lu R., Tian J., Zhang H., Zhou J., Sun M., Cao X., Zhu X., Liu B., Yu Q. (2026). The residue 86 of the Getah virus E2 glycoprotein mediates both glycosaminoglycan-and LDLR-dependent infection. PLoS Pathog..

[23] Contigiani M.S., Diaz L.A. (2016). Togaviridae. Arthropod Borne Diseases.

[24] Shen J., Liu S., Liu S., Shen S., Lei M., Xu Q., Li W., He Q., Xu X., Cai X. (2025). Genomic surveillance and evolution of Getah virus. Virus Evol..

[25] Yu L., Chen L. (2026). The epidemiological and evolutionary dynamics of Getah virus in China. Vet. Vaccine.

[26] Mou C., Meng H., Shi K., Huang Y., Liu M., Chen Z. (2023). GETV nsP2 plays a critical role in the interferon antagonism and viral pathogenesis. Cell Commun. Signal..

[27] Wang M., Sun Z., Cui C., Wang S., Yang D., Shi Z., Wei X., Wang P., Sun W., Zhu J. (2022). Structural insights into alphavirus assembly revealed by the cryo-EM structure of Getah virus. Viruses.

[28] Sun Z., Wang M., Wang W., Li D., Wang J., Sui G. (2024). Getah virus capsid protein undergoes co-condensation with viral genomic RNA to facilitate virion assembly. Int. J. Biol. Macromol..

[29] Meng H., Mou C., Zhang L., Zhou J., Lu T., Chen Z. (2023). The roles of 6K protein on Getah virus replication and pathogenicity. J. Med. Virol..

[30] Kim A.S., Diamond M.S. (2023). A molecular understanding of alphavirus entry and antibody protection. Nat. Rev. Microbiol..

[31] Uchime O., Fields W., Kielian M. (2013). The role of E3 in pH protection during alphavirus assembly and exit. J. Virol..

[32] Xing C., Jiang J., Lu Z., Mi S., He B., Tu C., Liu X., Gong W. (2020). Isolation and characterization of Getah virus from pigs in Guangdong province of China. Transbound. Emerg. Dis..

[33] Jones R., Bragagnolo G., Arranz R., Reguera J. (2021). Capping pores of alphavirus nsP1 gate membranous viral replication factories. Nature.

[34] Zhao M., Ji L., Zhao Y., Yang S., Yang H., He B., Wang S., Zhang W. (2026). Getah virus non-structural protein 2 inhibits type I interferon production by preventing K63-linked polyubiquitination of IKKε and causing widespread cellular shutoff. Microbiol. Spectr..

[35] Lark T., Keck F., Narayanan A. (2018). Interactions of alphavirus nsP3 protein with host proteins. Front. Microbiol..

[36] Qi X., Zhao R., Yao X., Liu Q., Liu P., Zhu Z., Tu C., Gong W., Li X. (2024). Getah virus Nsp3 binds G3BP to block formation of bona fide stress granules. Int. J. Biol. Macromol..

[37] Tan Y.B., Lello L.S., Liu X., Law Y.S., Kang C., Lescar J., Zheng J., Merits A., Luo D. (2022). Crystal structures of alphavirus nonstructural protein 4 (nsP4) reveal an intrinsically dynamic RNA-dependent RNA polymerase fold. Nucleic Acids Res..

[38] Qiu Z., Zhang D., Fan Q., Hu Z., Xie H., Li N., Xiao P. (2026). Getah Virus in Review: An Emerging Zoonotic Arbovirus. Transbound. Emerg. Dis..

[39] Wang N., Zhai X., Li X., Wang Y., He W.T., Jiang Z., Veit M., Su S. (2022). Attenuation of Getah Virus by a Single Amino Acid Substitution at Residue 253 of the E2 Protein that Might Be Part of a New Heparan Sulfate Binding Site on Alphaviruses. J. Virol..

[40] Elmasri Z., Nasal B.L., Jose J. (2021). Alphavirus-Induced Membrane Rearrangements during Replication, Assembly, and Budding. Pathogens.

[41] Zimmerman O., Holmes A.C., Kafai N.M., Adams L.J., Diamond M.S. (2023). Entry receptors—The gateway to alphavirus infection. J. Clin. Investig..

[42] Wang N., Merits A., Veit M., Lello L.S., Kong S., Jiao H., Chen J., Wang Y., Dobrikov G., Rey F.A. (2024). LDL receptor in alphavirus entry: Structural analysis and implications for antiviral therapy. Nat. Commun..

[43] Negi V., Miller A.S., Kuhn R.J. (2025). Advances in Viroporin Function and Structure: A Comparative Analysis of Alphavirus 6K with Well-Characterized Viroporins. Viruses.

[44] Wang S., Mahalingam S., Merits A. (2025). Alphavirus nsP2: A Multifunctional Regulator of Viral Replication and Promising Target for Anti-Alphavirus Therapies. Rev. Med. Virol..

[45] Liu H., Qi Z., Tian L., Chen Z., Li H., Liu L., Liu S., Li S., Sun J., Shao Y. (2025). Getah virus nonstructural protein 2 suppresses interferon-beta production by interrupting interferon regulatory factor 3 activation. Vet. Res..

[46] Zhai X., Li X., Veit M., Wang N., Wang Y., Merits A., Jiang Z., Qin Y., Zhang X., Qi K. (2024). LDLR is used as a cell entry receptor by multiple alphaviruses. Nat. Commun..

[47] Tanaka A., Tumkosit U., Nakamura S., Motooka D., Kishishita N., Priengprom T., Sa-Ngasang A., Kinoshita T., Takeda N., Maeda Y. (2017). Genome-Wide Screening Uncovers the Significance of N-Sulfation of Heparan Sulfate as a Host Cell Factor for Chikungunya Virus Infection. J. Virol..

[48] Mohamed-Romai-Noor N.A., Sam S.S., Teoh B.T., Hamim Z.R., AbuBakar S. (2022). Genomic and In Vitro Phenotypic Comparisons of Epidemic and Non-Epidemic Getah Virus Strains. Viruses.

[49] Shi N., Zhu X., Qiu X., Cao X., Jiang Z., Lu H., Jin N. (2022). Origin, genetic diversity, adaptive evolution and transmission dynamics of Getah virus. Transbound. Emerg. Dis..

[50] Ren T., Liu M., Li P., Zhang L., Zhong Z., Zhou L., Qin Y., Ouyang K., Yin Y., Chen Y. (2026). Defining the impact of Getah virus envelope protein glycosylation site mutations on viral replication, host adaptation, virulence, and immune evasion. PLoS Pathog..

[51] Fros J.J., Pijlman G.P. (2016). Alphavirus Infection: Host Cell Shut-Off and Inhibition of Antiviral Responses. Viruses.

[52] Liu Y., Yuan Y., Zhang L. (2022). Innate immune evasion by alphaviruses. Front. Immunol..

[53] Li J., Gao X., Liu X., Wu T., Song H., Gao W., Jia H., Li Y., Zhang Z. (2023). The host transcriptome change involved in the inhibitory effect of exogenous interferon-γ on Getah virus replication. Front. Microbiol..

[54] Liu Y., Yao Z., Zhang Y., Zhu Z., Wang Z., Chen Q., Yang Z., He Y., Chen X., Tian L. (2026). Alphaviral non-structural protein-host RBP co-condensation as a mechanism to sustain virus replication. Mol. Cell.

[55] Lu G., Chen R., Shao R., Dong N., Liu W., Li S. (2020). Getah virus: An increasing threat in China. J. Infect..

[56] Lu G., Ou J., Ji J., Ren Z., Hu X., Wang C., Li S. (2019). Emergence of Getah Virus Infection in Horse with Fever in China, 2018. Front. Microbiol..

[57] Takeishi M., Kuwata R., Ono T., Sasaki A., Ogata M., Iwata E., Taji S., Koike M., Nemoto M., Bannai H. (2022). Seroconversion of anti-Getah virus antibody among Japanese native Noma horses around 2012. J. Vet. Med. Sci..

[58] Wu Y., Gao X., Kuang Z., Lin L., Zhang H., Yin L., Hong J., Ren B., Li Q., Wang L. (2024). Isolation and pathogenicity of a highly virulent group III porcine Getah virus in China. Front. Cell. Infect. Microbiol..

[59] Zhao B., Du B., Wang J., Zhang Z., Yang D., He X., Wang J. (2026). Establishment of a mouse model for studying emerging Getah virus pathogenesis and vaccine efficacy. J. Integr. Agric..

[60] Xiao P., Hao Y., Zheng M., Zhang D., Li N. (2025). The first discovery of the group IV GETV from Pangolin, China. Virulence.

[61] Shi N., Qiu X., Cao X., Mai Z., Zhu X., Li N., Zhang H., Zhang J., Li Z., Shaya N. (2022). Molecular and serological surveillance of Getah virus in the Xinjiang Uygur Autonomous Region, China, 2017–2020. Virol. Sin..

[62] Rawle D.J., Nguyen W., Dumenil T., Parry R., Warrilow D., Tang B., Le T.T., Slonchak A., Khromykh A.A., Lutzky V.P. (2020). Sequencing of Historical Isolates, K-mer Mining and High Serological Cross-Reactivity with Ross River Virus Argue against the Presence of Getah Virus in Australia. Pathogens.

[63] Azerigyik F.A., Faizah A.N., Kobayashi D., Amoa-Bosompem M., Matsumura R., Kai I., Sasaki T., Higa Y., Isawa H., Iwanaga S. (2023). Evaluating the mosquito host range of Getah virus and the vector competence of selected medically important mosquitoes in Getah virus transmission. Parasit Vectors.

[64] Deng Y.L., Lin Z.Y., Lin R., Lu Z.S., Yan X.X., Li L.X., Lu H.J., Bai X., Jin N.Y., Liu H. (2025). Isolation and Characterization of Getah Virus GD2202 from Mosquitoes in Foshan, China. Vector Borne Zoonotic Dis..

[65] Ke Yee C., Lani R., Hassandarvish P. (2026). The oxidative stress paradigm in arbovirus infections: Mechanisms and therapeutic insights. Redox Rep..

[66] McAllister N., Liu Y., Silva L.M., Lentscher A.J., Chai W., Wu N., Griswold K.A., Raghunathan K., Vang L., Alexander J. (2020). Chikungunya Virus Strains from Each Genetic Clade Bind Sulfated Glycosaminoglycans as Attachment Factors. J. Virol..

[67] Zhao J., Dellicour S., Yan Z., Veit M., Gill M.S., He W.-T., Zhai X., Ji X., Suchard M.A., Lemey P. (2023). Early genomic surveillance and phylogeographic analysis of Getah virus, a reemerging arbovirus, in livestock in China. J. Virol..

[68] Cai R., He Q., Wang Q., Tian L., Chen Z., Wu X., Sun J., Shao Y., Song X., Qi K. (2025). Development of a reverse genetics system for Getah virus and characterization of rescued strains. Vet. Res..

[69] Li F., Deng L., Xu T., Xu L., Xu Z., Lai S., Ai Y., Wang Y., Yan G., Zhu L. (2025). Getah virus triggers ROS-mediated autophagy in mouse Leydig cells. Front. Microbiol..

[70] Kril V., Hons M., Amadori C., Zimberger C., Couture L., Bouery Y., Burlaud-Gaillard J., Karpov A., Ptchelkine D., Thienel A.L. (2024). Alphavirus nsP3 organizes into tubular scaffolds essential for infection and the cytoplasmic granule architecture. Nat. Commun..

